# *﻿Ceriporiopsistianshanensis* (Polyporales, Agaricomycetes) and *Sideratianshanensis* (Hymenochaetales, Agaricomycetes), two new species of wood-inhabiting fungi from Xinjiang, Northwest China

**DOI:** 10.3897/mycokeys.98.102552

**Published:** 2023-05-29

**Authors:** Tai-Min Xu, Yi-Fei Sun, Shun Liu, Chang-Ge Song, Neng Gao, Dong-Mei Wu, Bao-Kai Cui

**Affiliations:** 1 Institute of Microbiology, School of Ecology and Nature Conservation, Beijing Forestry University, Beijing 100083, China Beijing Forestry University Beijing China; 2 Xinjiang Production and Construction Group Key Laboratory of Crop Germplasm Enhancement and Gene Resources Utilization, Biotechnology Research Institute, Xinjiang Academy of Agricultural and Reclamation Sciences, Shihezi, Xinjiang 832000, China Biotechnology Research Institute, Xinjiang Academy of Agricultural and Reclamation Sciences Shihezi China

**Keywords:** macrofungi, phylogeny, polyporoid fungi, taxonomy, white-rot fungi

## Abstract

Wood-inhabiting fungi are abundant in China, but their distribution is uneven, with more fungi in southwest China and fewer fungi in northwest China. During the investigation of wood-inhabiting fungi in Xinjiang, we collected a large number of specimens. Eight specimens growing on *Piceaschrenkiana* were collected from Tianshan Mountains, and they were described as two new species in *Ceriporiopsis* and *Sidera* based on morphological characters and molecular evidence. *Ceriporiopsistianshanensis* is characterized by a cream to salmon-buff pore surface, larger pores measuring 1–3 per mm, and broadly ellipsoid basidiospores 5–6.5 × 3–4 μm. *Sideratianshanensis* is characterized by annual to perennial basidiocarps, measuring 15 mm thick, pores 5–7 per mm, cream to rosy buff pore surface, and allantoid basidiospores 3–3.5 × 1–1.4 µm. Detailed illustrations and descriptions of the novel species are provided.

## ﻿Introduction

China is rich in wood-inhabiting fungal resources, and more than 2000 species of the woody fungi have been reported (Dai 2010, 2012; Cui et al. 2019; Wu et al. 2022a, b). In the past ten years, many new species of the wood-inhabiting fungi have been discovered in China, and mainly distributed in the southwest and south areas, and few new species have been published from northwest China (Li et al. 2014; Chen et al. 2016; Shen et al. 2019; Wang et al. 2021; Yuan et al. 2021; Ji et al. 2022; Wu et al. 2022b; Liu et al. 2023a).

The Xinjiang Uygur Autonomous Region is located in northwestern China, and, as the largest province in China, it covers an area of 1,664,900 square kilometers. There is a typical temperate continental arid climate, with an extremely uneven distribution of water resources in time and space, more in the west and less in the east, more in the north and less in the south, more in the mountains and less in the plains (Wu et al. 2010; Hu et al. 2021). Due to severe climatic conditions, natural forests are mainly distributed in the Tianshan Mountains and Altai Mountains (Zhang and Zhang 2014; Huang et al. 2018). In the past, 592 species of macrofungi have been reported in Xinjiang, among which 243 are species of wood rot fungi, and most of them were distributed in the Tianshan Mountains and the Altai Mountains (Wang and Ayinuer 2004; Dai et al. 2007; Bau et al. 2008; Guli et al. 2015; Zhao 2022). In recent years, some new species of wood rot fungi have been discovered in Xinjiang: *Fomitopsistianshanensis* B.K. Cui & Shun Liu, *Laetiporusxinjiangensis* J. Song, Y.C. Dai & B.K. Cui, *Porodaedaleaschrenkianae* Y.C. Dai & F. Wu, and *Rhodoniatianshanensis* Yuan Yuan & L.L. Shen (Song et al. 2014; Yuan and Shen 2017; Liu et al. 2021a; Wu et al. 2022b).

During the investigation of wood rot fungi in Xinjiang, we collected a large number of specimens, including two belonging to *Ceriporiopsis* and six belonging to *Sidera*. The genus *Ceriporiopsis* Domański (Meruliaceae, Polyporales) was erected by Domański (1963) based on the morphological analyses to accommodate *C.gilvescens* (Bres.) Domański (type species), *C.incarnata* Domański, *C.resinascens* (Romell) Domański, *C.aneirina* (Sommerf.) Domańsk and *C.placenta* (Fr.) Domański. Currently, there are 41 species accepted in *Ceriporiopsis*, and eight species recorded in China: *C.albonigrescens* Núñez, Parmasto & Ryvarden, *C.aurantitingens* (Corner) T. Hatt., *C.egula* C.J. Yu & Y.C. Dai, *C.lavendula* B.K. Cui, *C.micropora* T.T. Chang & W.N. Chou, *C.mucida* (Pers.) Gilb. & Ryvarden, *C.subrufa* (Ellis & Dearn.) Ginns and *C.subsphaerospora* (A. David) M. Pieri & B. Rivoire (Zhao and Cui 2014; Zhao et al. 2015, 2023). The genus causes a white rot on angiosperms and gymnosperms (Niemelä 1985; Zhao and Cui 2014; Zhao et al. 2015; Spirin and Ryvarden 2016). It is characterized by annual, resupinate to effused-reflexed basidiocarps, a monomitic hyphal system with no action in Melzer’s reagent or Cotton Blue, generative hyphae with clamp connections, and subcylindrical to ellipsoid basidiospores whih hyaline, thin walls (Gilbertson and Ryvarden 1987; Núñez and Ryvarden 2001; Ryvarden and Melo 2014; Zhao and Wu 2017). In phylogenetic analysis, *Ceriporiopsis* was polyphyletic and clustered into the phlebioid clade (Zhao and Cui 2014; Zhao et al. 2015; Zhao and Wu 2017). Zmitrovich (2018) transferred *C.gilvescens* and *C.kunmingensis* to the genus *Mycoacia* Donk (Zmitrovich 2018). Zhao et al. (2023) conducted a detailed phylogenetic analysis, and many species within *Ceriporiopsis* were placed in the genera *Ceriporiopsoides* C.L. Zhao, *Hydnophlebia* Parmasto, and *Phlebicolorata* C.L. Zhao. The remaining *Ceriporiopsis* species did not belong to the phlebioid clade but were grouped in the residual polyporoid clade and formed a relatively stable branch cluster. The genus *Sidera* Miettinen & K.H. Larss. (Rickenellaceae, Hymenochaetales) was established by Miettinen and Larsson (2011) based on phylogenetic and morphological analyses to accommodate *S.lunata* (Romell ex Bourdot & Galzin) K.H. Larss., *S.lowei* (Rajchenb.) Miettinen, *S.lenis* (P. Karst.) Miettinen (type species) and *S.vulgaris* (Fr.) Miettinen. To date, 18 species are accepted in *Sidera*, nine species were recorded in China: *S.borealis* Z.B. Liu & Yuan Yuan, *S.inflata* Z.B. Liu & Y.C. Dai, *S.lenis*, *S.minutissima* Y.C. Dai, F. Wu, G.M. Gates & Rui Du, *S.parallela* Y.C. Dai, F. Wu, G.M. Gates & Rui Du, *S.punctata* Z.B. Liu & Y.C. Dai, *S.roseobubalina* Z.B. Liu & Y.C. Dai, *S.salmonea* Z.B. Liu, Jian Yu & F. Wu, *S.tibetica* Z.B. Liu, Jian Yu & F. Wu (Liu et al. 2023b). The genus causes a white rot in the wood, and is characterized by resupinate basidiocarps that are white to cream or buff, mostly waxy when fresh, with a poroid or hydnoid hymenophore, monomitic or dimitic hyphal system with generative hyphae bearing clamp connections, the presence of rosette-like crystals and allantoid to lunate basidiospores (Miettinen and Larsson 2011; Du et al. 2020; Liu et al. 2021b, 2022). In phylogenetic analysis, *Sidera* is a monophyletic genus and clustered into the *Rickenella* clade (Liu et al. 2021b, 2022, 2023b). In this study, two new species are described based on morphological and phylogenetic evidence.

## ﻿Materials and methods

### ﻿Morphological studies

The specimens used in this study were deposited at the herbarium of the Institute of Microbiology, Beijing Forestry University, China (BJFC). Macro-morphological descriptions were based on field notes and laboratory measurements. The microscopic routines used in this study followed Cui et al. (2019) and Liu et al. (2023a). Sections were studied at a magnification up to ×1000 using a Nikon E80i microscope and phase contrast illumination (Nikon, Tokyo, Japan). Line drawings were made with the aid of a drawing tube. Microscopic features, measurements and drawings were made from slide preparations of dried or fresh material stained with Cotton Blue and Melzer’s reagent, as described by Dai (2010). To represent the variation in the size of the basidiospores, 5% of measurements were excluded from each end of the range and are given in parentheses. The following abbreviations were used: IKI = Melzer’s reagent, IKI–= neither dextrinoid nor amyloid, KOH = 5% potassium hydroxide, CB = Cotton Blue, CB–= acyanophilous, L = mean spore length (arithmetic average of all spores), W = mean spore width (arithmetic average of all spores), Q = variation in the L/W ratios between the specimens studied, n = number of spores measured from a given number of specimens. Color terms followed Petersen (1996).

### ﻿DNA extraction and sequencing

Total genomic DNA was extracted from dried specimens using a cetyltrime-thylammonium bromide (CTAB) Rapid Plant Genome Extraction Kit (Aidlab Biotech-nologies Company, Ltd., Beijing, China) according to the manufacturer’s instructions with some modifications (Li et al. 2014; Ji et al. 2022). Two DNA gene fragments, ITS and nLSU, were amplified using the primer pairs ITS5/ITS4 and LR0R/LR7 (White et al. 1990). The PCR procedures for ITS and nLSU followed Song et al. (2022) and Sun et al. (2022) in the phylogenetic analyses. All PCR products were directly purified and sequenced at the Beijing Genomics Institute (BGI), China, with the same primers. Newly generated sequences were submitted to GenBank and are listed in Tables 1, 2.

**Table 1.** List of species, specimens and GenBank accession numbers of sequences used in the phylogeny of *Ceriporiopsis*.

Species	Sample no.	Location	GenBank accession no.		Reference
			ITS	nLSU	
*Ceriporiopsisandreanae*	CBS 279.92	USA, Montana	ALYI01000630	–	Cheng et al. 2022
*C.herbicola*	K 132752	UK, Oxfordshire	KX008364	KX081076	Zhao and Wu 2017
*C.pseudogilvescens*	Niemelä 7447	Finland	FJ496680	FJ496700	Tomšovský et al. 2010
*C.pseudogilvescens*	TAA 168233	Estonia	FJ496673	FJ496702	Tomšovský et al. 2010
*C.pseudogilvescens*	BRNM 686416	Slovakia	FJ496679	FJ496703	Tomšovský et al. 2010
*C.subrufa*	BRNM 710164	Czech Republic	FJ496661	FJ496723	Tomšovský et al. 2010
*C.subrufa*	BRNM 710172	Czech Republic	FJ496662	FJ496724	Tomšovský et al. 2010
*C.tianshanensis*	Cui 19150	China, Xinjiang	OP920992	OP920984	Present study
*C.tianshanensis*	Cui 19151	China, Xinjiang	OP920993	OP920985	Present study
*Ceriporiopsoidesguidella*	HUBO 7659	Italy	FJ496687	FJ496722	Tomšovský et al. 2010
*C.lagerheimii*	58240	Ecuador, Napo	KX008365	KX081077	Zhao and Wu 2017
*Hydnophlebiafimbriata*	Dai 11672	China, Hunan	KJ698633	KJ698637	Zhao et al. 2015
*H.fimbriata*	Cui 1671	China, Jiangsu	KJ698634	KJ698638	Zhao et al. 2015
*Mycoaciagilvescens*	BRNM 710166	Czech Republic	FJ496684	FJ496720	Tomšovský et al. 2010
*M.gilvescens*	Yuan 2752	China, Shaanxi	KF845953	KF845946	Zhao and Cui 2014
*M.gilvescens*	BRNM 667882	Czech Republic	FJ496685	FJ496719	Tomšovský et al. 2010
*M.kunmingensis*	C.L. Zhao 152	China, Yunnan	KX081072	KX081074	Zhao and Wu 2017
*M.kunmingensis*	C.L. Zhao 153	China, Yunnan	KX081073	KX081075	Zhao and Wu 2017
*Phlebicolorataalboaurantia*	Cui 2877	China, Fujian	KF845954	KF845947	Zhao and Cui 2014
*P.alboaurantia*	Cui 4136	China, Fujian	KF845948	KF845955	Zhao and Cui 2014
*P.pseudoplacenta*	JV 050952	USA, Tennessee	JN592499	JN592506	Vlasák et al. 2012
*P.pseudoplacenta*	PRM 899297	USA	JN592497	JN592504	Vlasák et al. 2012
*P.rosea*	Dai 13573	China, Yunnan	KJ698635	KJ698639	Zhao et al. 2015
*P.rosea*	Dai 13584	China, Yunnan	KJ698636	KJ698640	Zhao et al. 2015
*P.semisupina*	Cui 10222	China, Zhejiang	KF845949	KF845956	Zhao and Cui 2014
*P.semisupina*	Cui 10189	China, Zhejiang	KF845958	KF845951	Zhao and Cui 2014
*P.semisupina*	Cui 7971	China, Yunnan	KF845950	KF845957	Zhao and Cui 2014
*Raduliporusaneirinus*	Dai 12657	Finland, Helsinki	KF845952	KF845945	Zhao and Cui 2014
*Antrodiaserpens*	Dai 7465	Luxemburg	KR605813	KR605752	Liu et al. 2023a
*Rhodofomesroseus*	Cui 17046	China, Yunnan	ON417187	ON417238	Liu et al. 2023a

**Table 2.** List of species, specimens and GenBank accession numbers of sequences used in the phylogeny of *Sidera*.

Species	Sample no.	Location	GenBank accession no.		Reference
			ITS	nLSU	
*Siderainflata*	Cui 13610	China, Hainan	MW198480	–	Liu et al. 2021b
*S.lenis*	Miettinen 11036	Finland	FN907914	FN907914	Miettinen and Larsson 2011
*S.lunata*	JS 15063	Norway	DQ873593	DQ873593	Miettinen and Larsson 2011
*S.malaysiana*	Dai 18570	Malaysia	MW198481	MW192007	Liu et al. 2021b
*S.minutipora*	Gates FF257	Australia	FN907922	FN907922	Miettinen and Larsson 2011
*S.minutipora*	Cui 16720	Australia	MN621349	MN621348	Du et al. 2020
*S.minutissima*	Dai 18471A	China, Hainan	MW198482	MW192008	Liu et al. 2021b
*S.minutissima*	Dai 19529	Sri Lanka	MN621352	MN621350	Du et al. 2020
*S.parallela*	Cui 10346	China, Yunnan	MK346145	–	Du et al. 2020
*S.parallela*	Cui 10361	China, Yunnan	MK346144	–	Du et al. 2020
*S.punctata*	Dai 22119	China, Hainan	MW418438	MW418437	Liu et al. 2021b
*S.roseo-bubalina*	Dai 11277	China, Henan	MW198483	–	Liu et al. 2021b
*S.salmonea*	Dai 23354	China, Tibet	OM974250	OM974242	Liu et al. 2022
*S.salmonea*	Dai 23343	China, Tibet	OM974249	OM974241	Liu et al. 2022
*S.salmonea*	Dai 23428	China, Tibet	OM974251	OM974243	Liu et al. 2022
***S.tianshanensis***	**Cui 19132**	**China, Xinjiang**	**OP920994**	**OP920986**	**Present study**
***S.tianshanensis***	**Cui 19143**	**China, Xinjiang**	**OP920995**	**OP920987**	**Present study**
***S.tianshanensis***	**Cui 19186**	**China, Xinjiang**	**OP920996**	**OP920988**	**Present study**
***S.tianshanensis***	**Cui 19192**	**China, Xinjiang**	**OP920997**	**OP920989**	**Present study**
***S.tianshanensis***	**Cui 19196**	**China, Xinjiang**	**OP920998**	**OP920990**	**Present study**
***S.tianshanensis***	**Cui 19251**	**China, Xinjiang**	**OP920999**	**OP920991**	**Present study**
*S.srilankensis*	Dai 19654	Sri Lanka	MN621344	MN621346	Du et al. 2020
*S.srilankensis*	Dai 19581	Sri Lanka	MN621345	MN621347	Du et al. 2020
*S.tenuis*	Dai 18698	Australia	MK331866	MK331868	Du et al. 2020
*S.tenuis*	Dai 18697	Australia	MK331865	MK331867	Du et al. 2020
*S.tibetica*	Dai 23648	China, Tibet	OM974253	OM974245	Liu et al. 2022
*S.tibetica*	Dai 23407	China, Tibet	OM974252	OM974244	Liu et al. 2022
*S.tibetica*	Dai 21057	Belarus	MW198484	MW192009	Liu et al. 2021b
*S.tibetica*	Dai 22151	China, Guangxi	MW477794	MW474965	Liu et al. 2021b
*S.vesiculosa*	BJFC025367	Singapore	MH636565	MH636567	Du et al. 2020
*S.vesiculosa*	BJFC025377	Singapore	MH636564	MH636566	Du et al. 2020
*S.borealis*	Cui 11216	China, Shaanxi	MW198485	–	Liu et al. 2021b
*S.vulgaris*	Ryvarden 37198	New Zealand	FN907918	FN907918	Miettinen and Larsson 2011
*S.lowei*	Miettinen X419	Venezuela	FN907917	FN907917	Miettinen and Larsson 2011
*S.lowei*	Miettinen X426	New Zealand	FN907919	FN907919	Miettinen and Larsson 2011
*Skvortzoviafurfuraceum*	KHL 11738	Finland	DQ873648	DQ873648	Liu et al. 2022
*S.furfurella*	KHL 10180	Puerto Rico	DQ873649	DQ873649	Liu et al. 2022

### ﻿Phylogenetic analysis

Phylogenetic analyses for *Ceriporiopsis* and *Sidera* were performed with maximum parsimony (MP), maximum likelihood (ML) and Bayesian inference (BI) analyses based on the combined ITS+nLSU dataset. New generated sequences were aligned with the additional sequences retrieved from GenBank (Tables 1, 2) using BioEdit 7.0.5.3 (Hall 1999) and ClustalX 1.83 (Thompson et al. 1997), followed by manual adjustments. *Antrodiaserpens* (Fr.) P. Karst. and *Rhodofomesroseus* (Alb. & Schwein.) Kotl. & Pouzar were used as outgroups in the phylogeny of *Ceriporiopsis* (Zhao and Wu 2017), while *Skvortzoviafurfurella* (Bres.) Bononi & Hjortstam and *Skvortzoviafurfuracea* (Bres.) G. Gruhn & Hallenberg were used as outgroups in the phylogeny of *Sidera* (Liu et al. 2022).

Maximum parsimony (MP) analysis was performed in PAUP* version 4.0b10 (Swofford 2002). The settings for phylogenetic analyses in this study followed the approach of Ji et al. (2022) and Zhu et al. (2019). All characters were equally weighted, and gaps were treated as missing data. Trees were inferred using the heuristic search option with TBR branch swapping and 1000 random sequence additions. Max trees were set to 5000, branches of zero length were collapsed, and all parsimonious trees were saved. Clade robustness was assessed using a bootstrap (BT) analysis with 1000 replicates (Felsenstein 1985). Descriptive tree statistics tree length (TL), consistency index (CI), retention index (RI), rescaled consistency index (RC) and homoplasy index (HI) were calculated for each generated Maximum Parsimonious Tree (MPT) (Page 1996).

Maximum likelihood (ML) analysis was conducted by RAxML-HPC252 through the CIPRES Science Gateway (www.phylo.org) and involved 100 ML searches. All model parameters were estimated by the program. Only the best maximum likelihood tree from all searches was retained. The maximum likelihood bootstrap values (ML-BS) were determined using rapid bootstrapping with 1000 replicates. The phylogenetic tree was visualized using Treeview (Page 1996).

Bayesian inference (BI) analysis was implemented in MrBayes 3.2.6 (Ronquist et al. 2012). There were two independent runs, each of which had four chains for 1,000,000 generations sampling from the posterior distribution every 1000^th^ generation to check that the PSRF (potential scale reduction factors) were reasonably close to 1.0 for all parameters indicative of chain convergence. The first 25% of the sampled trees were discarded as burn-in, while the remaining trees were used to obtain the Bayesian posterior probabilities (BPPs) of the clades. A majority rule consensus tree of all remaining trees was calculated.

Branches that received bootstrap support for maximum parsimony (MP), maximum likelihood (ML) higher than or equal to 75% (MP and ML-BS) and Bayesian posterior probabilities (BPP) higher than or equal to 0.95 (BPP) were considered significantly supported. The best topologies from MP analyses are shown in this study, and the final alignments and the retrieved topologies were deposited in TreeBASE (http://www.treebase.org accessed on 28 April 2023), under accession ID: 29931.

## ﻿Results

### ﻿Molecular phylogeny

**Figure 1.**
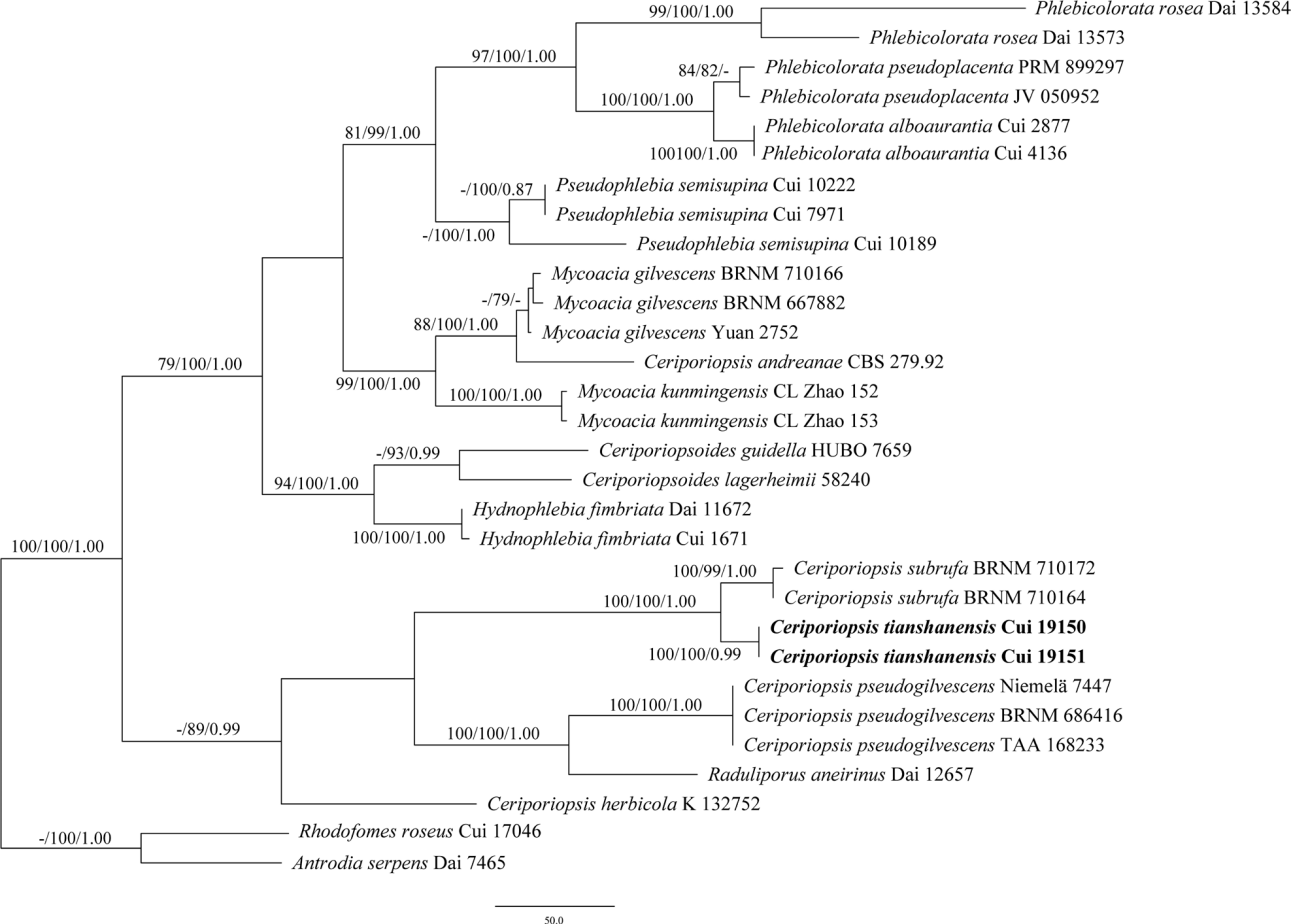
Maximum parsimony (MP) tree of *Ceriporiopsis* based on the combined ITS+nLSU dataset. Branches are labelled with maximum parsimony/maximum likelihood bootstrap values higher than 75% and Bayesian posterior probability values greater than 0.95. The new species is indicated in bold.

The phylogeny of *Ceriporiopsis*, based on a combined ITS and nLSU dataset, included 30 ITS sequences and 29 nLSU sequences from 30 fungal specimens, representing 17 species. The dataset had an aligned length of 2153 characters, of which 1399 characters were constant, 200 were variable and parsimony-uninformative and 554 were parsimony informative. Maximum parsimony analysis yielded one equally parsimonious tree (TL = 1902, CI = 0.601, RI = 0.763, RC = 0.459, HI = 0.399), and a strict consensus tree of these trees is shown in Fig. 1. The best model fit applied in the Bayesian inference analysis was GTR+I+G. Bayesian analysis and ML analysis resulted in a similar topology to MP analysis, with an average standard deviation of split frequencies of 0.006591 (BI).

**Figure 2.**
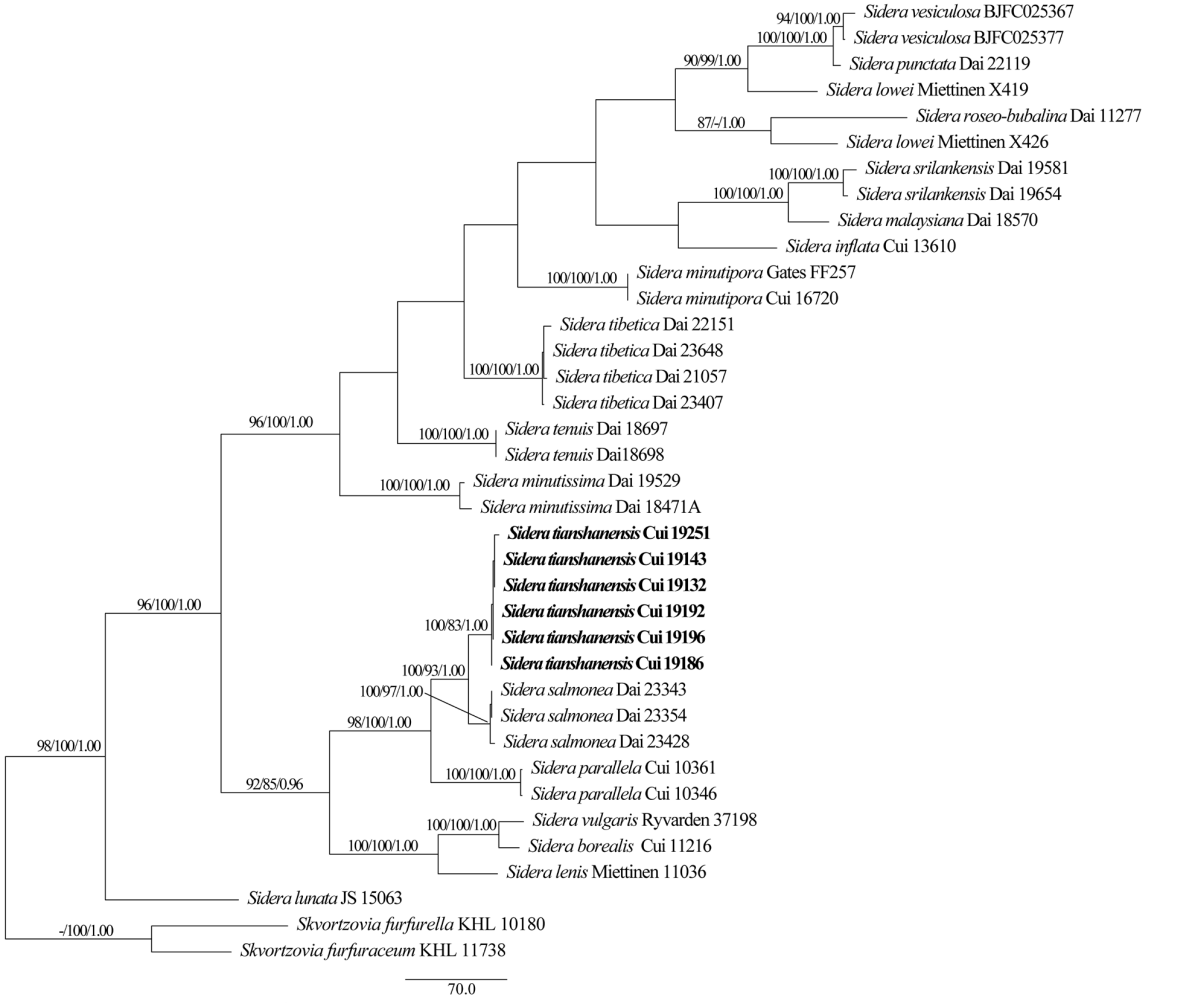
Maximum parsimony (MP) tree of *Sidera* based on the combined ITS+nLSU dataset. Branches are labelled with maximum parsimony/maximum likelihood bootstrap values higher than 75% and Bayesian posterior probabilities greater than 0.95.

The phylogeny of *Sidera*, based on a combined ITS and nLSU dataset, included 37 ITS sequences and 32 nLSU sequences from 37 fungal specimens, representing 19 species. The dataset had an aligned length of 2235 characters, of which 1453 characters were constant, 205 were variable and parsimony-uninformative and 577 were parsimony in-formative. Maximum parsimony analysis yielded one equally parsimonious tree (TL = 2233, CI = 0.583, RI = 0.760, RC = 0.443, HI = 0.417), and a strict consensus tree of these trees is shown in Fig. 2. The best model fit applied in the Bayesian inference analysis was GTR+I+G. Bayesian analysis and ML analysis resulted in a similar topology as MP analysis, with an average standard deviation of split frequencies of 0.007516 (BI).

Within the phylogenetic tree of *Ceriporiopsis*, the new species *C.tianshanensis* was closely related to *C.subrufa* with high supports (100% ML, 100% MP, 1.00 BPP; Fig. 1). However, the ITS sequences of *Ceriporiopsistianshanensis* and *C.subrufa* were significantly different, with 31 different nucleobases, and the similarity was 94.80% by nucleotide blast. The difference in the nLSU sequence was not significant; there were 4 different nucleobases, and the similarity was 99.29% by nucleotide blast.

In addition, the phylogenetic tree of *Sidera*, the new species *Sideratianshanensis*, was closely related to *S.salmonea* with high support (100% ML, 93% MP, 1.00 BPP; Fig. 2). However, the ITS sequences of *Sideratianshanensis* and *S.salmonea* were significantly different, with 40 different nucleobases, and the similarity was 94.29% by nucleotide blast. The difference in the nLSU sequence was not significant; there were 7 different nucleobases, and the similarity was 99.55% by nucleotide blast.

### ﻿Taxonomy

#### ﻿ Ceriporiopsis tianshanensis

Taxon classificationFungiPolyporalesPhanerochaetaceae

B.K. Cui & T.M. Xu sp. nov.

5A8384CD-1E13-53E4-B4B7-AFA15BCA0B4A

848610

Figs 3 , 4

##### Diagnosis.

*Ceriporiopsistianshanensis* is characterized by a cream to salmon-buff pore surface when fresh, large pores measuring 1–3 per mm, broadly ellipsoid basidiospores measuring 5–6.5 × 3–4 μm, and growth on the stump of *Piceaschrenkiana* Fisch. et Mey.

##### Type.

China. Xinjiang Autonomous Region, Tekes County, Kosang Cave National Forest Park, on the stump of *Piceaschrenkiana*, 19 September 2021, Cui 19150 (holotype).

##### Etymology.

tianshanensis (Lat.): referring to the species occurrence in Tianshan.

##### Fruiting body.

Basidiocarps annual, resupinate, adnate, not easily separated from the substrate, soft corky when fresh, fragile to hard fibrous when dry, up to 12 cm long, 3 cm wide, 2 mm thick. Pore surface white to cream or salmon-buff when fresh, becoming buff to vinaceous-buff or fawn when dry; pores irregular, 1–3 per mm; dissepiments thin, entire. Subiculum cream to buff and fibrous to soft corky when dry, up to 4 mm thick. Tubes concolorous with pore surface, corky, up to 4 mm long.

##### Hyphal structure.

Hyphal system monomitic; generative hyphae with clamp connections, lack crystal, IKI–, CB–; tissues unchanged in KOH.

##### Subiculum.

Generative hyphae hyaline, thin- to slightly thick-walled, often branched, interwoven, 3.5–5 μm in diameter.

##### Tubes.

Generative hyphae hyaline, thin- to slightly thick-walled, occasionally branched, interwoven, 3–6 μm in diameter. Cystidia and other sterile hymenial elements absent. Basidia short clavate to barrel-shaped, bearing four sterigmata and a basal clamp connection, 12–22 × 5–6 μm; basidioles dominant, in shape similar to basidia, but smaller.

**Figure 3.**
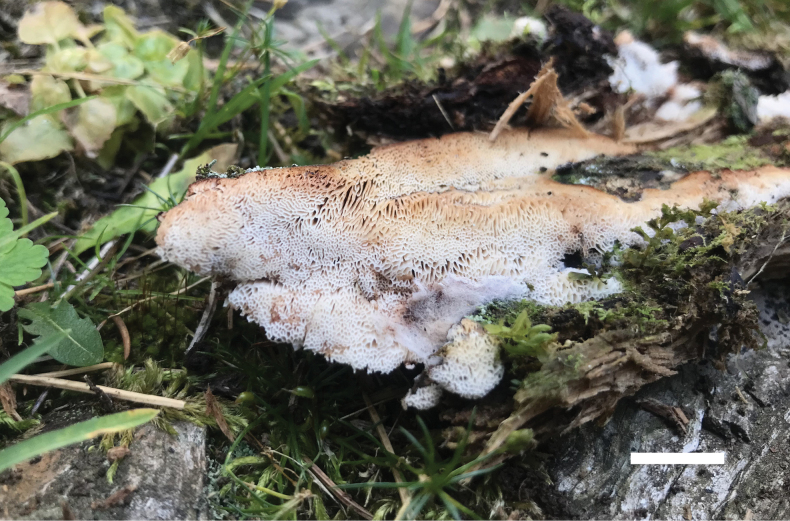
Basidiocarps of *Ceriporiopsistianshanensis* (Cui 19151). Scale bar: 1.0 cm.

##### Spores.

Basidiospores broadly ellipsoid, colorless, thin-walled, smooth, often with one guttule, IKI–, CB–, 5–6.5 × 3–4 µm, L = 5.9 µm, W = 3.5 µm, Q = 1.69–1.74 (n = 60/2).

**Figure 4.**
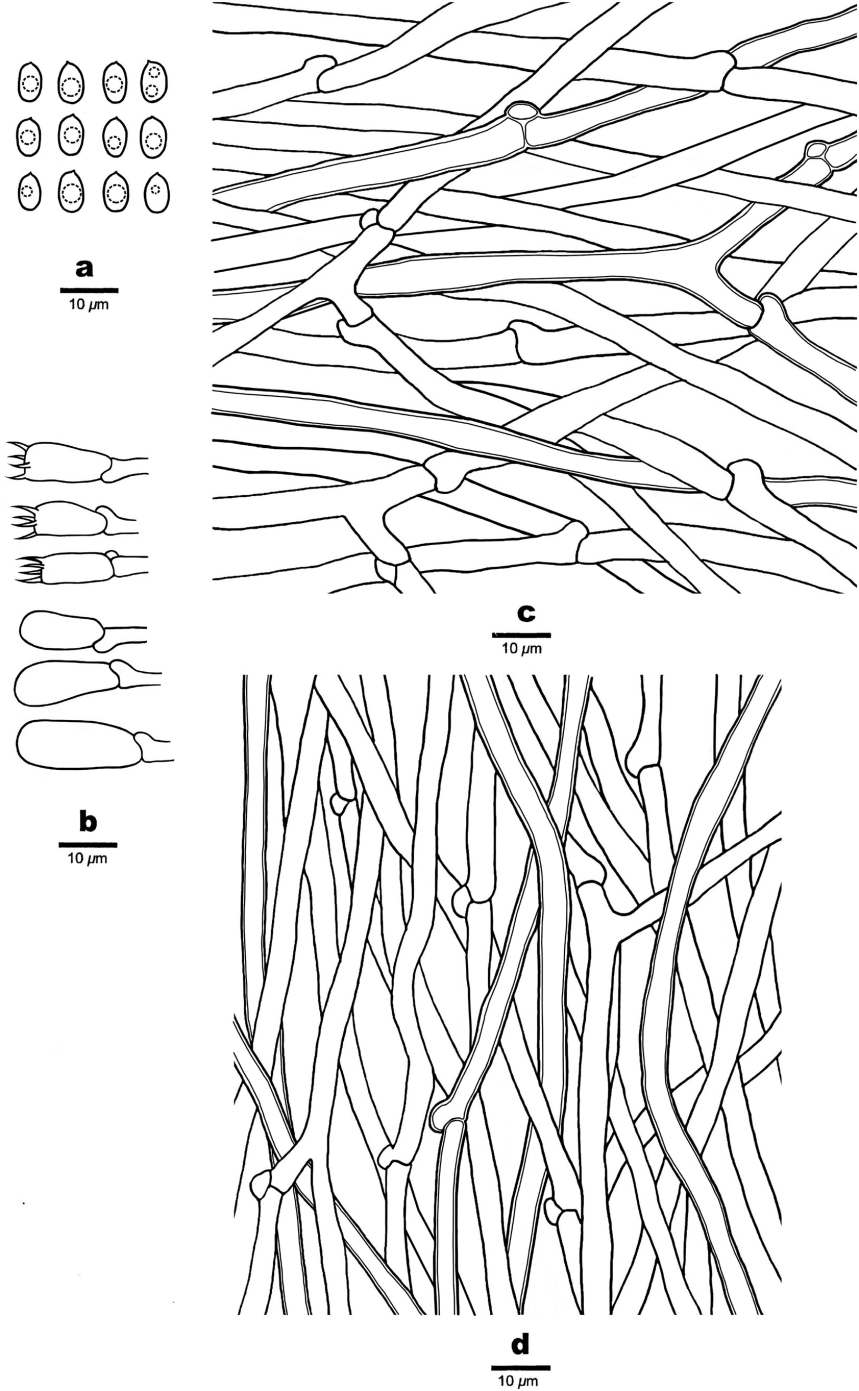
Microscopic structures of *Ceriporiopsistianshanensis* (Cui 19150) **a** basidiospores **b** basidia and basidioles **c** hyphae from the subiculum **d** hyphae from trama.

##### Type of rot.

White rot.

##### Additional specimen (paratype) examined.

China. Xinjiang Autonomous Region, Tekes County, Kosang Cave National Forest Park, on the stump of *Piceaschrenkiana*, 19 September 2021, Cui 19151.

#### ﻿ Sidera tianshanensis

Taxon classificationFungiHymenochaetalesCantharellaceae

B.K. Cui & T.M. Xu sp. nov.

C39C4AEE-8330-532F-9584-3A2657BE849F

848611

Figs 5 , 6

##### Diagnosis.

*Sideratianshanensis* is characterized by annual to perennial basidiocarps, measuring 15 mm thick, pores measuring 5–7 per mm, cream to rosy buff pore surface, allantoid basidiospores measuring 3–3.5 × 1–1.4 µm, and growing on the stump or trunk of *Piceaschrenkiana*.

**Figure 5.**
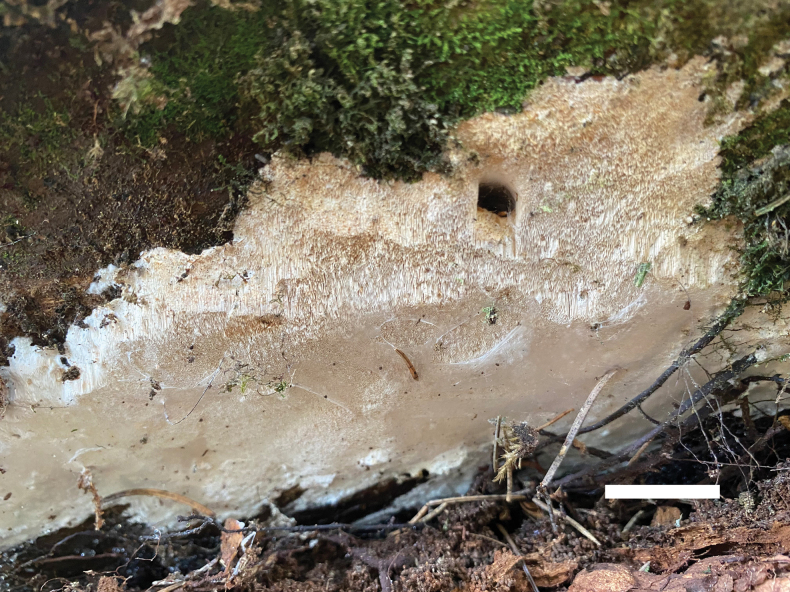
Basidiocarp of *Sideratianshanensis* (Cui 19143). Scale bar: 2.0 cm.

##### Type.

China. Xinjiang Autonomous Region, Tekes County, Kosang Cave National Forest Park, on fallen trunk of *Piceaschrenkiana*, 19 September 2021, Cui 19143 (holotype).

**Figure 6.**
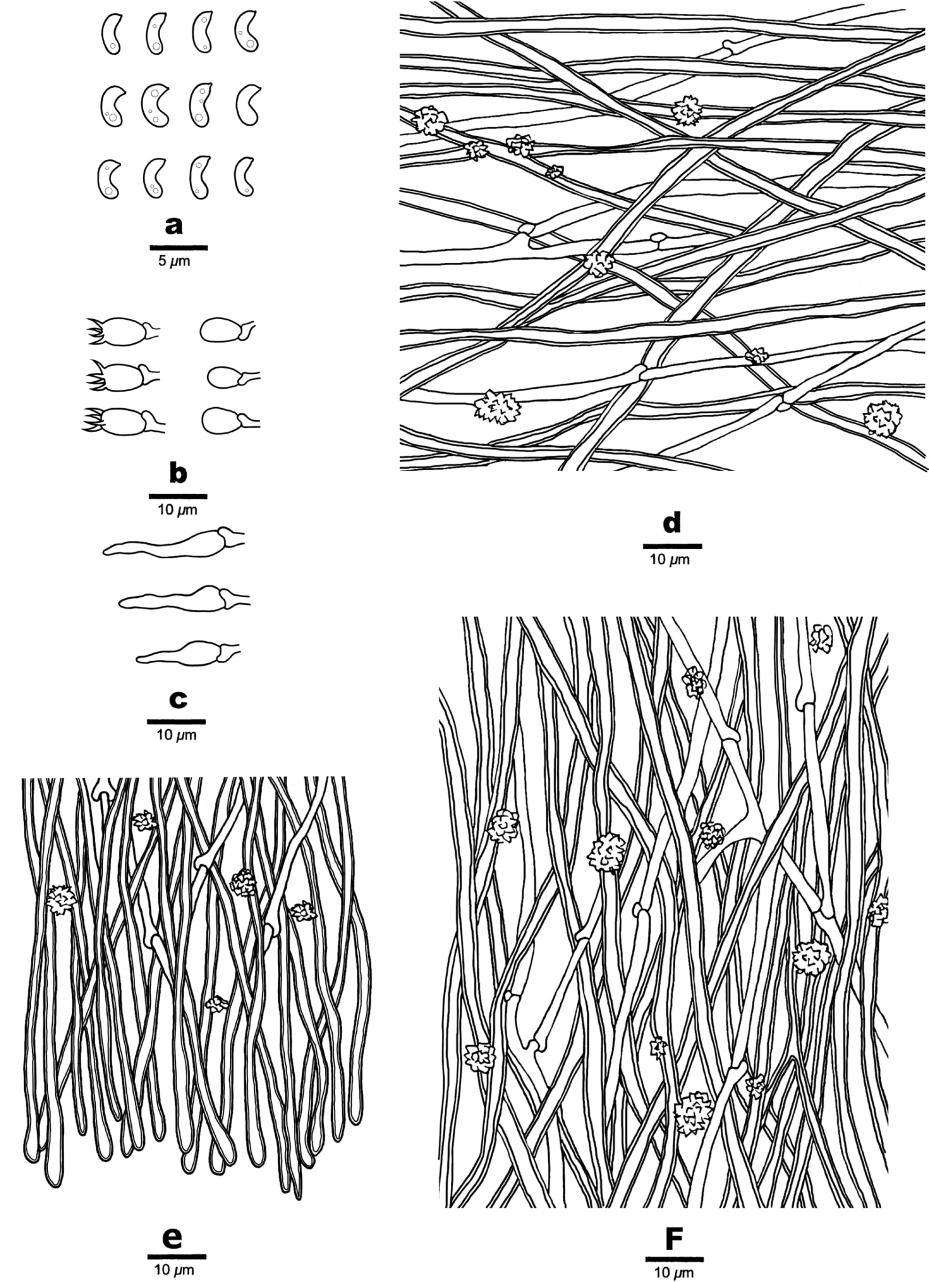
Microscopic structures of *Sideratianshanensis* (Cui 19143) **a** basidiospores **b** basidia and basidioles **c** Cystidioles **d** hyphae from the subiculum **e** hyphae at the disappearance edge **f** hyphae from trama.

##### Etymology.

tianshanensis (Lat.): referring to the species occurrence in Tianshan.

##### Fruiting body.

Basidiocarps annual to perennial, resupinate, soft corky, up to 10 cm long, 5 cm wide, and 15 mm thick at the center; pore surface cream to buff yellow, uncracked; sterile margin indistinct, cottony, white, thinning out; pores angular, 5–7 per mm; dissepiments thin, entire; subiculum white, cottony and up to 0.1 mm thick; tubes concolorous with pore surface, up to 15 mm long.

##### Hyphal structure.

Hyphal system dimitic; generative hyphae bearing clamp connections; rosette-like crystals frequently present; all hyphae IKI–, CB–; tissue unchanged in KOH.

##### Subiculum.

Generative hyphae infrequent, thin-walled, hyaline, occasionally branched, 2–2.5 µm in diameter; skeletal hyphae dominant, interwoven, unbranched, 2–3 µm diameter.

##### Tubes.

Generative hyphae infrequent, thin-walled, hyaline, occasionally branched, 1.5–2.5 µm in diameter; skeletal hyphae dominant, thick-walled with a wide to medium lumen, hyaline, occasionally branched, interwoven, flexuous, 2–3 µm in diameter. Cystidia absent; cystidioles present, fusoid, thin-walled, hyaline, basally swollen, with hyphoid neck and sharp tip, 15–22 × 3–4 µm. Basidia barrel-shaped, hyaline, bearing four sterigmata and a basal clamp connection, 5.5–7 × 3.5–4.5 µm; basidioles pyriform, shorter than the basidia.

##### Spores.

Basidiospores allantoid, hyaline, thin-walled, smooth, occasionally with one or more guttules, IKI–, CB–, 3–3.5 × 1–1.4 µm, L = 3.12 µm, W = 1.18 µm, Q = 2.6–2.7 (n = 150/5).

##### Type of rot.

White rot.

##### Additional specimens (paratypes) examined.

China. Xinjiang Autonomous Region, Tekes County, Kosang Cave National Forest Park, on stump of *Piceaschrenkiana*, 19 September 2021, Cui 19132; Tekes County, Karada Town, Qiongkushitai Village, on stump of *Piceaschrenkiana*, 19 September 2021, Cui 19186, Cui 19192; on fallen trunk of *Piceaschrenkiana*, 19 September 2021, Cui 19196, Cui 19251.

## ﻿Discussion

In this study, phylogenetic trees of *Ceriporiopsis* and *Sidera* were constructed using combined ITS and nLSU sequences, respectively. The two newly proposed species formed separate branches on the phylogenetic trees with high support. In addition, both *Ceriporiopsistianshanensis* and *Sideratianshanensis* differ from other recorded species through their morphological characteristics.

According to our phylogenetic analyses of *Ceriporiopsis* based on the combined ITS+nLSU dataset, *Ceriporiopsistianshanensis* was involved in *Ceriporiopsis* s.s. with strong support (100% ML, 100% MP, 1.00 BPPs) (Fig. 1). *Ceriporiopsissubrufa* was closely related to *C.tianshanensis* in the phylogenetic tree (Fig. 1), but there were obvious morphological differences between them. *Ceriporiopsissubrufa* is distinguished from *C.tianshanensis* by thicker basidiocarps (10 mm) and angular pores, and by growing on angiosperm trees (Ryvarden and Gilbertson 1993). Mophologically, *Ceriporiopsistianshanensis* is similar to *C.pseudoplacenta* Vlasák & Ryvarden and *C.aneirina* (Sommerf.) Domański by white to cream to salmon-buff pore surface, and by broadly ellipsoid basidiospores of similar size (Ryvarden and Gilbertson 1993; Vlasák et al. 2012). However, *Ceriporiopsispseudoplacenta* has thicker basidiocarps (6 mm), circular to angular pores and smaller basidiospores (3.5–4.5 × 2.2–3 µm) (Vlasák et al. 2012). The difference between *Ceriporiopsisaneirina* and *C.tianshanensis* is that the former has thicker basidiocarps (4 mm), generative hyphae with thin walls and crystals, and grows on angiosperm trees (Ryvarden and Gilbertson 1993).

The phylogenetic analysis of *Sidera* showed that *Sideratianshanensis* was involved in *Sidera* s.s. with strong support (100% ML, 96% MP, 1.00 BPPs) (Fig. 2). In addition, *Siderasalmonea* was closely related to *S.tianshanensis* in the phylogenetic tree (Fig. 2). However, *Siderasalmonea* is distinguished from *S.tianshanensis* by its shape and size of the basidiospores (3–3.5 × 0.9–1.1 µm, Q = 3.03–3.21, lunate vs. 3–3.5 × 1–1.4 µm, Q = 2.6–2.7, allantoid), smaller pores (7–9 per mm vs. 5–7 per mm), and surface of basidiocarps being salmon to slightly shiny, while that of the latter is cream to rosy buff (Liu et al. 2022). Morphologically, *Sideratianshanensis* is similar to *S.parallela*, both presenting cream to buff yellow pore surfaces and the pore sizes were similar (Du et al. 2020). However, *Sideraparallela* differs from *S.tianshanensis* by its thinner basidiocarps (1.5 mm vs. 15 mm); in addition, *S.parallela* grows on angiosperm trees (Du et al. 2020), while *S.tianshanensis* grows on *Piceaschrenkiana*.

Based on the records in previous literature and the introduction in this study, 42 species of *Ceriporiopsis* have been recorded in the world, among which 9 species are distributed in China (Binder et al. 2005; Zhao and Wu 2017; Ryvarden 2018, 2019, 2020; Zhao et al. 2023). *Ceriporiopsis* is widely distributed across five continents, with the exception of Antarctica and Oceania. The genus is most diverse in Africa, where it is represented by 17 species. South America has 10 species, North America has 7 species, Asia has 7 species, and Europe has 4 species. A total of 19 species of *Sidera* have been recorded worldwide, among which 10 species are distributed in China (Liu et al. 2023b). *Sidera* is currently a genus of fungi that has been relatively understudied. Among the discovered species that have been discovered so far, Asia has the highest number with 13 species, followed by Oceania with 4 species, Europe with 3 species, and North and South America with 1 species each. With the in-depth investigation of wood-inhabiting fungi in Xinjiang, an increasing number of new species of wood-inhabiting fungi will be discovered. The species diversity of wood-inhabiting fungi in China will also be richer.

## Supplementary Material

XML Treatment for Ceriporiopsis tianshanensis

XML Treatment for Sidera tianshanensis

## ﻿Additional information

### Author ORCIDs

Tai-Min Xu https://orcid.org/0000-0002-5230-4140

Yi-Fei Sun https://orcid.org/0000-0003-3997-3662

Shun Liu https://orcid.org/0000-0001-9261-4365

Chang-Ge Song https://orcid.org/0000-0001-5379-2353

Neng Gao https://orcid.org/0009-0000-4745-987X

Dong-Mei Wu https://orcid.org/0009-0006-4126-0767

Bao-Kai Cui https://orcid.org/0000-0003-3059-9344

### Data availability

All of the data that support the findings of this study are available in the main text or Supplementary Information.
